# Supplementary material for: Allopatric Lineage Divergence of the East Asian Endemic Herb Conandron ramondioides Inferred from Low-Copy Nuclear and Plastid Markers
Source: Int J Mol Sci. 2022 Nov 29;23(23):14932. doi: 10.3390/ijms232314932 (PMC9740071; doi:10.3390/ijms232314932)
Supplement: Supplementary file 1 [file ijms-23-14932-s001.zip › ijms-2013506-supplementary.pdf]

Supplementary Table S1. Collection localities, haplotypes, genetic indices, neutrality test (including Tajima’s D and Fu’s Fs) obtained from 21 *C. ramondioides* collection sites.

			AGT intron1					GroES intron1					LEAFY intron1				
Region	Location		Hd	π ×10[-3]	Haplo-types (No. seq)	Tajima's D	Fu's Fs	Hd	π ×10[-3]	Haplo-types (No. seq)	Tajima's D	Fu's Fs	Hd	π ×10[-3]	Haplo-types (No. seq)	Tajima's D	Fu's Fs
Honshu	Hitachiota (Ryujin Waterfall), Ibaraki	HRI	0.8 ±0.0148	5.66	A01(2), A02(2), A03(2)	1.218	1.574	0.667 ±0.042	4.6	G01(2), G02(2)	2.012	2.197	0	0	L01(6)	-	-
	Hitachiota (Some-kawa Rivier), Ibaraki	HIS	0	0	A02(18)	-	-	0.662 ±0.002	15.05	G02(9), G03(9), G04(4)	1.785	**10.154**	0.312 ±0.011	0.79	L01(18), L02(4)	0.236	0.648
	Tsukuba, Ibaraki	TI	0.733 ±0.024	3.96	A01(1), A02(2), A03(3)	-0.825	0.893	0.933 ±0.015	18.54	G01(1), G03(1), G04(2), G05(1), G06(1)	0.518	0.461	0.607 ±0.026	2.36	L01(2), L02(5), L03(1)	-0.812	0.071
	Ichikai, Tochigi	IT	0.8 ±0.029	5.66	A01(1), A02(3), A03(1), A04(1)	0.084	0.022	0.467 ±0.017	4.29	G01(3), G07(7)	1.229	**3.779**	1 ±0.25	5.09	L04(1), L05(1)	-	-
	Hachioji (Biwataki of Mt. Takao-san), Tokyo	HBT	0	0	A01(4)	-	-	0.774 ±0.003	9.73	G04(1), G07(7), G08(6), G09(2), G10(4)	0.829	3.155	0	0	L06(12)	-	-
	Matsuzaki, Shizuoka	MS	0	0	A05(2)	-	-	0.333 ±0.046	4.6	G07(1), G11(5)	-1.367	2.996	0	0	L07(6)	-	-
	Tatsuno, Nagano	TN	0.533 ±0.029	5.66	A01(2), A02(4)	1.219	**3.696**	0.857 ±0.012	17.26	G02(1), G03(1), G04(2), G06(1), G12(3)	0.256	1.629	0.733 ±0.024	5.6	L01(3), L02(1), L06(2)	1.387	1.199
Region average (Honshu)			0.586 ±0.004	4.64		1.027	2.375	0.89 ±0.0002	12.13		0.459	1.528	0.695 ±0.002	5.37		1.574	0.778
Shikoku	Naka-cho, Tokushima	NT	0.689 ±0.01	6.09	A03(5), A04(2), A06(3)	1.454	2.963	0.883 ±0.003	11.59	G09(2), G13(4), G14(2), G15(1), G16(4), G17(1), G18(1), G19(1)	0.105	0.014	0.747 ±0.006	2.91	L08(4), L10(6), L11(2), L12(2)	0.647	-0.128
	Yoshinokawa, Tokushima	YT	0.533 ±0.029	1.13	A06(4), A07(2)	0.85	0.625	0.333 ±0.046	3.84	G13(5), G14(1)	-1.337	2.593	0.733 ±0.024	2.37	L08(2), L09(3), L10(1)	0.311	-0.304
Region average (Shikoku)			0.725 ±0.0054	6.03		1.222	2.476	0.801 ±0.005	9.12		-0.343	0.073	0.784 ±0.003	3.35		0.484	-0.415
North group (Honshu+Shikoku)			0.736 ±0.0018	6.32		0.773	2.292	0.925 ±0.0001	13.05		-0.218	-1.558	0.823 ±0.0008	6.63		0.211	-1.938
Iriomote	Iriomote-jima (Yutsun-gawa River), Okinawa	YO	0.75 ±0.019	3.94	A08(1), A09(4), A10(1), A11(2)	-0.92	0.005	0.378 ±0.032	0.93	G20(1), G21(8), G22(1)	-1.401	**-1.164**	0.733 ±0.024	2.21	L13(3), L14(1), L15(2)	-0.05	-0.427
	Iriomote-jima (Urauchi), Okinawa	UO	0.882 ±0.0015	6.05	A11(3), A13(2), A14(4), A15(4), A16(2), A17(1)	0.306	-0.392	0.465 ±0.013	1.21	G20(2), G21(19), G23(2), G24(1), G25(2)	-1.346	**-2.55**	0	0	L13(10)	-	-
Region average (Iriomote)			0.905 ±0.0007	5.57		-0.564	-2.405	0.492 ±0.009	1.63		-1.68	**-3.92**	0.342 ±0.019	0.91		-1.037	-0.979

			CrCYC1					*ITS*					cpDNA (trnL-F + trnH-psbA)					
Region	Location		Hd	π ×10[-3]	Haplo-types (No. seq)	Tajima's D	Fu's Fs	Hd	π ×10[-3]	Haplo-types (No. seq)	Tajima's D	Fu's Fs	No. seq	Hd	π ×10[-3]	Haplo-types (No. seq)	Tajima's D	Fu's Fs
Honshu	Hitachiota (Ryujin Waterfall), Ibaraki	HRI	0.25 ±0.032	0.31	Y2(2), Y4(14)	-1.055	-0.182	0.75 ±0.01936	1.98	I02(8), I15(2), I24(2), I29(4)	0.204	-0.844	8	0.607 ±0.027	0.58	cp01(4),	-0.448	-0.478
	Hitachiota (Some-kawa Rivier), Ibaraki	HIS	0.667 ±0.042	0.82	Y2(4), Y4(4)	1.633	0.54	0.500 ±0.07031	0.82	I02(6), I15(2)	-0.612	0.172	4	0	0	cp01(4)	-	-
	Tsukuba, Ibaraki	TI	0.786 ±0.023	1.9	Y2(8), Y3(2), Y4(2) Y5(2), Y6(2)	-0.923	**-1.748**	0.250 ±0.03247	0.41	I02(2), I24(14)	-1.054	-0.182	8	0	0	cp01(8)	-	-
	Ichikai, Tochigi	IT	0.667 ±0.017	1.17	Y1(10), Y2(4), Y3(4)	0.975	0.245	0.889 ±0.00569	7.01	I02(4), I10(4), I15(4), I19(2), I22(2), I23(2)	0.05	0.028	10	0.833 ±0.016	2.74	cp01(9), cp02(1)	-1.612	-1.035
	Hachioji (Biwataki of Mt. Takao-san), Tokyo	HBT	0.842 ±0.003	3.38	Y5(4), Y7(4), Y8(10), Y9(8), Y10(2), Y11(4)	0.495	0.162	0.125 ±0.01133	0.82	I02(32)	**-1.831**	1.247	16	0	0	cp01(16)	-	-
	Matsuzaki, Shizuoka	MS	0.667 ±0.042	0.82	Y8(4), Y15(4)	1.633	0.54	0.667 ±0.04167	1.09	I02(4), I15(4)	1.633	0.54	4	0	0	cp03(5)	-	-
	Tatsuno, Nagano	TN	0.833 ±0.009	1.92	Y2(6), Y13(6), Y14(2), Y15(2)	0.231	-1.42	0	0	I24(16)	-	-	9	0	0	cp04(9)	-	-
Region average (Honshu)			0.787 ±0.0008	4.63		-0.616	-0.101	0.762 ±0.0007	2.22		-1.362	-4.788	58	0.482 ±0.004	0.61		-0.636	-0.25
Shikoku	Naka-cho, Tokushima	NT	0.709 ±0.019	1.71	Y3(10), Y17(4), Y18(2), Y19(4)	-1.288	-1.266	0	0	I21(20)	-	-	10	0.644 ±0.0103	0.65	cp05(11)	0.222	-0.046
	Yoshinokawa, Tokushima	YT	0.7 ±0.0478	8.15	Y20(6), Y21(2), Y22(2)	0.59	0.59	0	0	I21(10)	-	-	5	0	0	cp05(5)	-	-
Region average (Shikoku)			0.767 ±0.0063	3.07		-0.355	0.851	0	0		-	-	15	0.841 ±0.001	3.16		-1.143	-2.513
North group (Honshu+Shikoku)			0.897 ±0.00036	4.7		-0.961	-5.241	0.802 ±0.00067	4.43		-1.068	-1.724	73	0.633 ±0.002	2.49		0.559	3.783
Iriomote	Iriomote-jima (Yutsun-gawa River), Okinawa	YO	0.810 ±0.017	2.12	Y1(6), Y2(4), Y3(2), Y4(2)	0.239	-0.428	0.857 ±0.01880	6.15	I15(6), I16(2), I17(2), I18(2), I19(2)	0.799	-0.257	7	0.667 ±0.0255	1.23	cp06(3), cp07(4)	0.755	0.668
	Iriomote-jima (Urauchi), Okinawa	UO	0.600 ±0.046	2.06	Y1(14), Y5(4), Y6(2), Y7(4)	-1.337	0.688	0.533 ±0.02963	0.87	I19(14), I20(10)	0.851	1.152	12	0.506 ±0.025	0.72	cp07(9)	**-1.623**	**-2.1**
Region average (Iriomote)			0.719 ±0.011	2.12		-1.026	-2.01	0.821 ±0.00668	4.63		-0.091	-0.004	19	0.752 ±0.007	1.42		-1.171	**-2.802**

Supporting Table S1. cont.

Region	Location		AGT intron1					GroES intron1					LEAFY intron1					*CrCYC1*					*ITS*					cpDNA (trnL-F + trnH-psbA)					
			Hd	π ×10^{-3}	Haplo-types (No. seq)	Tajima's D	Fu's Fs	Hd	π ×10^{-3}	Haplo-types (No. seq)	Tajima's D	Fu's Fs	Hd	π ×10^{-3}	Haplo-types (No. seq)	Tajima's D	Fu's Fs	Hd	π ×10^{-3}	Haplo-types (No. seq)	Tajima's D	Fu's Fs	Hd	π ×10^{-3}	Haplo-types (No. seq)	Tajima's D	Fu's Fs	No. seq	Hd	π ×10^{-3}	Haplo-types (No. seq)	Tajima's D	Fu's Fs
Taiwan	Shuitian, Hsinchu	ShH	0.933 ±0.014	6.67	A09(1), A18(1), A19(2), A20(1), A21(1)	0.128	-1.2	0.833 ±0.049	2.71	G21(1), G26(2), G27(1)	0.592	-0.658	0.5 ±0.07	1.27	L16(1),	-0.612	0.172	0.909 ±0.003	2.24	Y1(6), Y5(4), Y6(2), Y7(4), Y8(4), Y9(2), Y10(2)	0.362	**-2.864**	0.530 ±0.01846	3.61	I01(8), I10(3), I11(1)	0.432	2.595	12	0.591 ±0.012	1.5	cp08(5), cp09(7)	1.15	1.967
	Smagus, Hsinchu	Sm H	0.758 ±0.0033	2.2	A09(7), A22(7), A23(3), A24(1), A25(2)	-0.233	-1.022	0.645 ±0.007	2.79	G28(1), G29(18), G30(2), G31(1)	-1.209	-0.535	0.61 ±0.005	1.78	L15(12), L17(7), L18(3)	0.63	0.525	0.200 ±0.024	0.25	Y5(18), Y8(2)	-1.112	-0.339	0.778 ±0.00822	2.94	I01(3), I12(2), I13(4), I14(1)	1.048	0.361	10	0	0	cp10(10)	-	-
	Manapan, Miaoli	MM	0.833 ±0.049	3.89	A09(1), A26(2), A27(1)	1.089	0.006	0.803 ±0.004	3.49	G21(4), G32(6), G33(2)	0.466	0.26	0.303 ±0.022	0.77	L15(10), L19(2)	-0.195	0.297	0.277 ±0.023	1.65	Y5(4), Y11(10), Y12 (2), Y13(4)	-0.219	-0.277	0.714 ±0.01606	1.87	I01(3), I02(3), I03(4)	-0.302	0.263	10	0.417 ±0.036	1.92	cp11(8),	**-1.61**	2.091
	Shanlinxi , Nantou	SN	0	0	A26(10)	-	-	0	0	G21(10)	-	-	0.742 ±0.007	2.43	L15(5), L20(4), L21(1), L22(2)	1.29	-0.719	0.639 ±0.016	0.89	Y1(10), Y2(2), Y4(8)	-0.064	-0.239	0.639 ±0.01583	2.18	I02(5), I04(1), I05(3)	0.794	0.909	10	0	0	cp11(8)	-	-
	Caoling, Yunlin	CY	0.626 ±0.104	2.67	A26(8), A28(3), A29(3)	1.004	1.355	0	0	G21(14)	-	-	0.6 ±0.016	2.1	L15(10), L21(1), L23(3), L24(1), L25(1)	-1.49	**-1.924**	0.556 ±0.027	0.75	Y1(4), Y2(12), Y3(2)	-0.583	-0.532	0.844 ±0.01060	4.36	I02(4), I05(1), I06(1), I07(1), I08(1), I09(1)	-0.708	-1.102	9	0.222 ±0.028	0.19	cp12(10)	-1.088	-0.263
	Meiling, Tainan	MT	0	0	A26(8)	-	-	0.644 ±0.023	1.95	G21(7), G32(3)	0.526	-1.287	0	0	L19(6)	-	-	0	0	Y1(10)	-	-	0	0	I02(10)	-	-	10	0.533 ±0.032	2.94	cp11(6)	**-2.019**	1.872
Region average (Taiwan)			0.764 ±0.002	4.05		-1.202	**-4.405**	0.678 ±0.002	2.52		-1.351	**-3.362**	0.67 ±0.003	2.32		-1.498	**-6.452**	0.829 ±0.001	2.21		-0.473	**-4.95**	0.799 ±0.00147	3.66		-1.309	**-4.557**	61	0.865 ±0.001	3.81		-1.109	-0.784
SE-China	Huang-Shan, Anhui	HA S	0	0	A30(4)	-	-	0	0	G34(4)	-	-	0	0	L26(4)	-	-	0	0	Y2(4)	-	-	0.900 ±0.02592	7.52	I29(2), I30(2), I31(4)	0.461	0.357	4	0.5000 ±0.070	0.43	cp13(3), cp14(1)	-0.612	0.172
	Qingliang feng, Zhejiang	QF Z	1 ±0.5	2.11	A30(1), A31(1)	-	-	0	0	G34(8)	-	-	0	0	L27(8)	-	-	0	0	Y1(12)	-	-	0.848 ±0.00344	4.11	I01(6), I02(6), I10(6), I15(4), I24(2)	0.333	0.331	12	0	0	cp15(12)	-	-
	Shouning, Fujiang	SF	0.52 ±0.066	1.67	A12(18), A30(9), A33(1)	-0.437	0.483	0.442 ±0.0076	2.84	G35(20), G36(7), G37(1)	1.447	2.134	0	0	L28(6)	-	-	0.607 ±0.009	2.72	Y3(10), Y4(30), Y5(2), Y6(2), Y7(6)	0.122	1.337	0.779 ±0.00324	3.24	I32(6), I33(2), I34(4), I35(16), I36(20), I37(2)	**-2.113**	-1.364	25	0.66 ±0.004	0.92	cp16(21)	-0.996	-1.73
	Longzi-Shan, Fujiang	LF	0	0	A32(24)	-	-	0	0	G38(24)	-	-	0	0	L27(4)	-	-	0	0	Y8(15)	-	-	0	0	I36(12)	-	-	15	0	0	cp11(14)	-	-
Group average (SE-China)			0.684 ±0.0008	2.16		-0.146	0.049	0.726 ±0.0007	4.19		1.639	2.042	0.623 ±0.005	5.02		1.305	3.337	0.818 ±0.0006	5.54		1.401	2.366	0.777 ±0.0026	3.93		-1.115	-4.11	56	0.821 ±0.0005	6.34		1.472	4.61
South group (T+I+C)			0.9 ±0.0001	5.64		-1.501	**-12.323**	0.818 ±0.0004	4.71		-1.305	**-7.304**	0.818 ±0.0008	6.15		-0.934	-3.888	0.937 ±0.0002	10.32		0.257	-0.66	0.839 ±0.0002	3.43		-0.998	-4.707		0.894 ±0.0001	3.95		0.093	0.655
ALL	Total		0.927 ±0.0005	7		-1.508	**-14.907**	0.916 ±0.0001	9.25		-1.461	**-15.968**	0.908 ±0.0001	7.4		-1.174	**-12.619**	0.951 ±0.0002	12.76		-0.157	-3.826	0.864 ±0.0001	3.44		-1.211	-10.259		0.954 ±0.00002	6.58		-1.508	**-14.376**

Supplementary Table S2. Sequences used to estimate substitution rate

	trnL-F	leafy	
HIS71	OP689760[a]	MH237973[a]	
Sma01	OP689848[a]	MH238036[a]	
CFS01	OP689918[a]	MH238072[a]	
Streptocarpus rexii	KR704015[b]	AY526321[b]	
[a] This study<br>[b] REF			

The clock rates of trnL-F, trnH-psbA and leafy for *C. ramondioides* were estimated based on the divergence between *C. ramondioides* and *Streptocarpus rexii*, both of which belonged to Didymocarpoideae. For each specific gene, we computed its clock rate, R, using the formula R = k/2T, where k and T stood for sequence divergence and divergence time between *C. ramondioides* and *S. rexii*, respectively. The T value was derived from Perret et al. (2012) who reconstructed a phylogeny for Gesneriaceae based on concatenated sequences of multiple cp markers and dated this tree with multiple fossil calibrations. In such a tree, the divergence time between *S. rexii* and *Primulina* sp. was estimated as 23 MYA (95% CI: 6.76-31.61 MYA). Given that *Conandron* and *Primulina* are phylogenetically closer relatives of each other to the exclusion of *Streptocarpus* (Möller et al., 2009; Weber et al., 2011), which necessitating the same divergence date between *Streptocarpus* and *Conandron* as between *Streptocarpus* and *Primulina*, we specified the T value as 23 MYA (95% CI: 6.76-31.61 MYA).

The k value for each of trnL-F, and leafy was calculated as mean sequence divergence between three *C. ramondioides* – *S. rexii* sequence pairs. We estimated sequence divergence using the Kimura-2 parameter model (Kimura 1980) in MEGA6 (Tamura et al., 2013). With k values thus obtained and the T value described above, we derived R values for trnL-F and leafy as 0.000935 (95% CI: 0.00068, 0.00318), and 0.0037 (95% CI: 0.0027, 0.0127) substitutions per nucleotide site per million years, respectively.

Supplementary Table S3. Numbers of sequenced *C. ramondioides ITS* haplotypes

	No. colony	No. haplotypes
QFZ03	5	4
LF15	6	3
SF14	6	6
CY10	6	3
MM07	6	6
SmH02	6	4
IT07	5	5
NT04	6	3

Supplementary Table S4. Pairwise genetic distance measured from *CrCYC1* dataset among assigned groups.

	J	TI	C
J	0		
TI	0.019	0	
C	0.016	0.018	0

Supplementary Table S5. Pairwise genetic distance measured from *ITS* dataset among assigned groups.

	J	TI	C
J	0		
TI	0.004	0	
C	0.003	0.004	0

A

		R	G	B
A1		0	32	96
A2		48	84	150
A3		142	169	219
A4		180	198	231
A5		217	225	242
A6		173	114	232
A7		121	81	163
A8		55	86	35
A9		84	130	53
A10		169	208	142
A11		198	224	180
A12		226	239	218
A13		44	250	44
A14		76	248	76
A15		108	249	108
A16		146	249	146
A17		174	250	174
A18		0	0	0
A19		128	128	128
A20		166	166	166
A21		191	191	191
A22		217	217	217
A23		242	242	242
A24		123	146	123
A25		101	131	101
A26		127	171	127
A27		81	107	81
A28		102	144	123
A29		25	115	121
A30		198	89	17
A31		244	176	132
A32		248	203	173
A33		255	0	0
A34		214	81	106
A35		216	107	127
A36		200	118	133

B

		R	G	B
G01		0	32	96
G02		48	84	150
G03		142	169	219
G04		180	198	231
G05		217	225	242
G06		173	114	232
G07		121	81	163
G08		55	86	35
G09		84	130	53
G10		169	208	142
G11		198	224	180
G12		226	239	218
G13		44	250	44
G14		76	248	76
G15		108	249	108
G16		146	249	146
G17		174	250	174
G18		0	0	0
G19		128	128	128
G20		166	166	166
G21		191	191	191
G22		217	217	217
G23		242	242	242
G24		123	146	123
G25		101	131	101
G26		127	171	127
G27		81	107	81
G28		102	144	123
G29		25	115	121
G30		198	89	17
G31		244	176	132
G32		248	203	173
G33		255	0	0
G34		214	81	106
G35		216	107	127
G36		200	118	133
G37		219	175	183
G38		196	169	174
G39		255	255	0
G40		224	224	134
G41		206	178	58
G42		201	183	105
G43		211	197	136
G44		247	201	11
G45		211	173	13
G46		175	143	11
G47		147	120	6
G48		102	84	33

C

		R	G	B
L01		0	32	96
L02		48	84	150
L03		142	169	219
L04		180	198	231
L05		217	225	242
L06		173	114	232
L07		121	81	163
L08		55	86	35
L09		84	130	53
L10		169	208	142
L11		198	224	180
L12		226	239	218
L13		44	250	44
L14		76	248	76
L15		108	249	108
L16		146	249	146
L17		174	250	174
L18		0	0	0
L19		128	128	128
L20		166	166	166
L21		191	191	191
L22		217	217	217
L23		242	242	242
L24		123	146	123
L25		101	131	101
L26		127	171	127
L27		81	107	81
L28		102	144	123
L29		25	115	121

D

		R	G	B
Y01		0	32	96
Y02		48	84	150
Y03		142	169	219
Y04		180	198	231
Y05		217	225	242
Y06		173	114	232
Y07		121	81	163
Y08		55	86	35
Y09		84	130	53
Y10		169	208	142
Y11		198	224	180
Y12		226	239	218
Y13		44	250	44
Y14		76	248	76
Y15		108	249	108
Y16		146	249	146
Y17		174	250	174
Y18		0	0	0
Y19		128	128	128
Y20		166	166	166
Y21		191	191	191
Y22		217	217	217
Y23		242	242	242
Y24		123	146	123
Y25		101	131	101
Y26		127	171	127
Y27		81	107	81
Y28		102	144	123
Y29		25	115	121
Y30		198	89	17
Y31		244	176	132
Y32		248	203	173
Y33		255	0	0
Y34		214	81	106
Y35		216	107	127
Y36		200	118	133
Y37		219	175	183
Y38		196	169	174
Y39		255	255	0
Y40		224	224	134
Y41		206	178	58
Y42		201	183	105
Y43		211	197	136
Y44		247	201	11
Y45		211	173	13
Y46		175	143	11
Y47		147	120	6
Y48		102	84	33

E

		R	G	B
I01		0	32	96
I02		48	84	150
I03		142	169	219
I04		180	198	231
I05		217	225	242
I06		173	114	232
I07		121	81	163
I08		55	86	35
I09		84	130	53
I10		169	208	142
I11		198	224	180
I12		226	239	218
I13		44	250	44
I14		76	248	76
I15		108	249	108
I16		146	249	146
I17		174	250	174
I18		0	0	0
I19		128	128	128
I20		166	166	166
I21		191	191	191
I22		217	217	217
I23		242	242	242
I24		123	146	123
I25		101	131	101
I26		127	171	127

F

		R	G	B
CP01		0	32	96
CP02		48	84	150
CP03		142	169	219
CP04		180	198	231
CP05		217	225	242
CP06		173	114	232
CP07		121	81	163
CP08		55	86	35
CP09		84	130	53
CP10		169	208	142
CP11		198	224	180
CP12		226	239	218
CP13		44	250	44
CP14		76	248	76
CP15		108	249	108
CP16		146	249	146
CP17		174	250	174
CP18		0	0	0
CP19		128	128	128

Supplementary Figure S1. Color codes for six molecular markers.

A: color codes for ATG2 intron 1 molecular marker, B: color codes for GroES intron 1 marker, C: color code for LEAFY marker, D: color code for CYC1 marker, E: color code for *ITS* marker, F: color code for chloroplast marler (trnL-F + trnH-psbA).

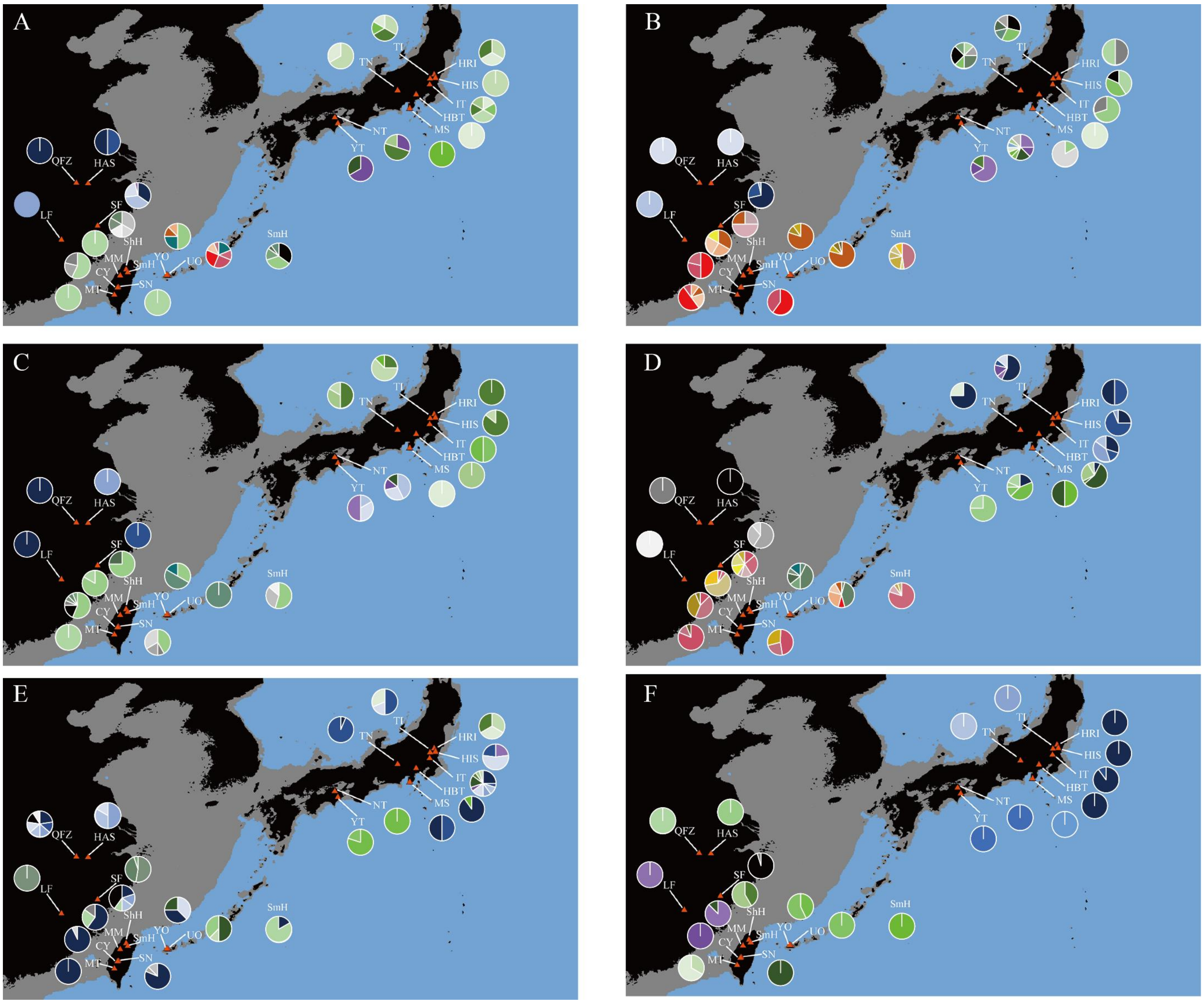

Supplementary Figure S2. Haplotype and ribotype frequency maps identified from the six molecular loci dataset.

A: frequency map of ATG2 intron 1 molecular marker, B: frequency map of GroES intron 1 marker, C: frequency map of LEAFY marker, D: frequency map of CYC1 marker, E: frequency map of *ITS* marker, F: frequency map of chloroplast marler (trnL-F + trnH-psbA).

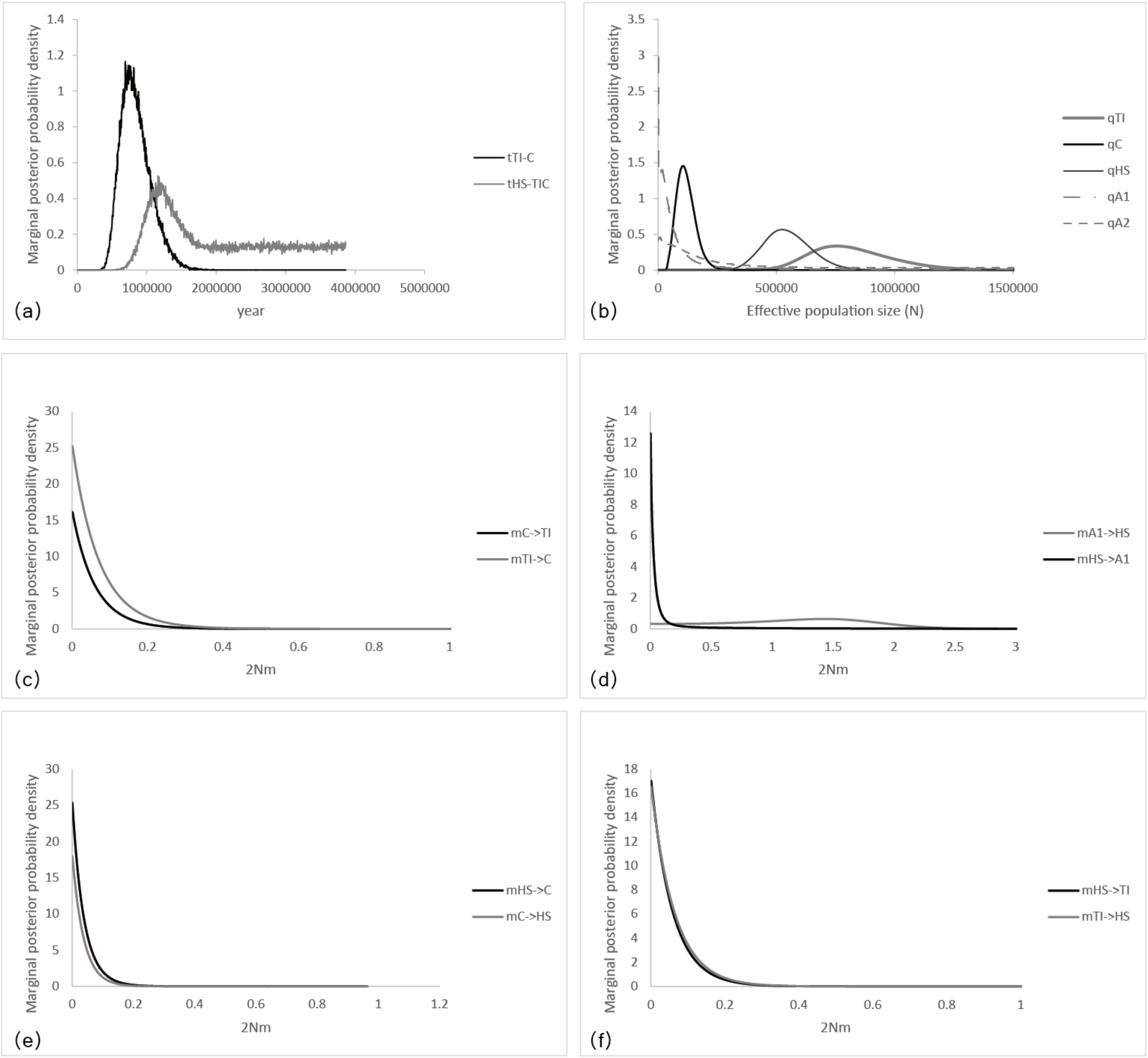

Supplementary Figure S3. Marginal posterior probability (MPP) curves obtained from multi-population IMa2 analysis.

A: MPP curves obtained from estimated divergence time parameters, B: MPP curves obtained from estimated effective population size parameters, C-F: MPP curves obtained from estimated post-divergence gene flow.

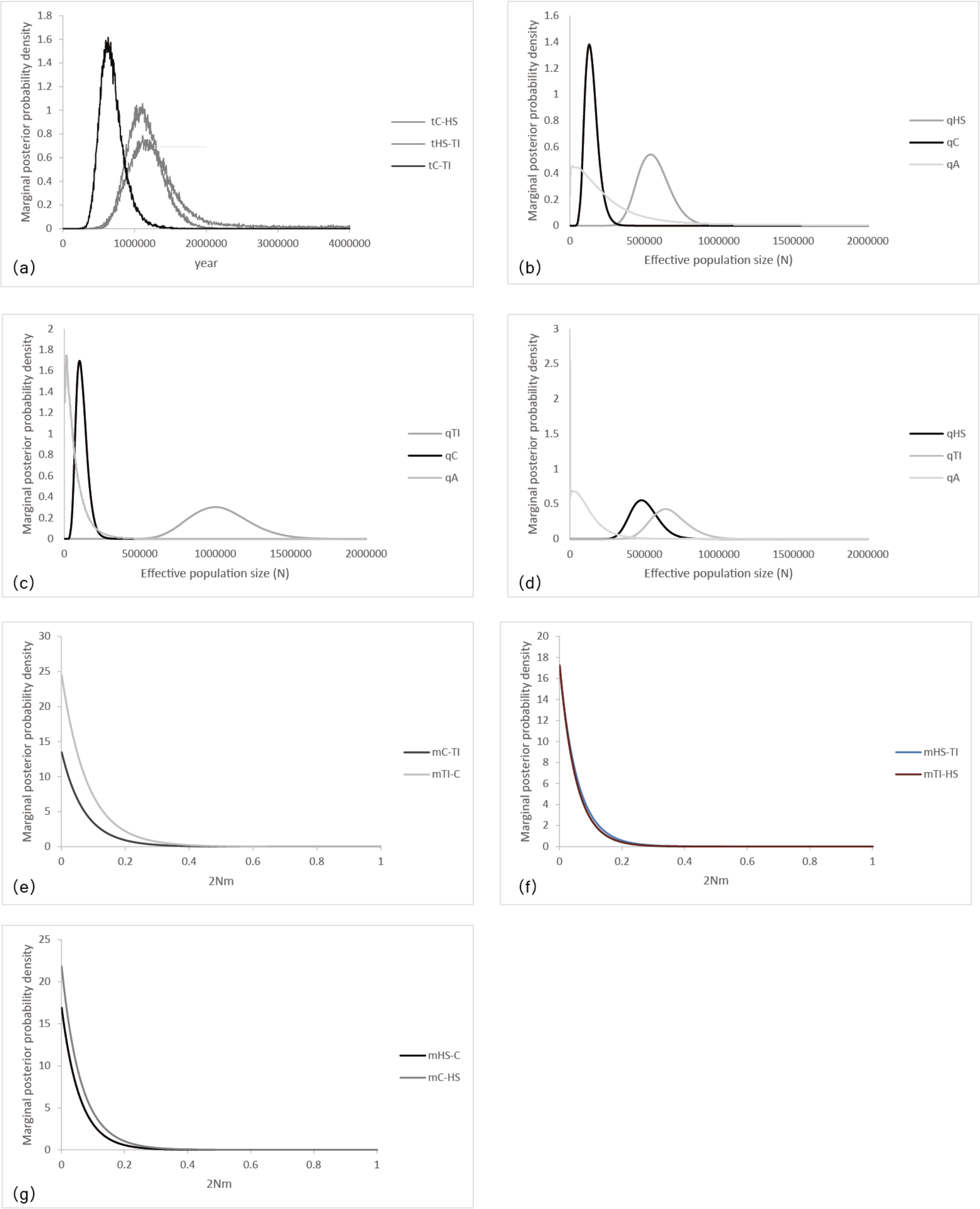

Supplementary Figure S4. Marginal posterior probability (MPP) curves obtained from pairwise IMa2 analysis.
A: MPP curves obtained from estimated divergence time parameters, B-D: MPP curves obtained from estimated effective population size parameters, E-G: MPP curves obtained from estimated post-divergence gene flow.
